# The impact of delayed placement and resin matrix composition on adhesion between layers of resin composites

**DOI:** 10.2340/biid.v13.46529

**Published:** 2026-07-16

**Authors:** Suvi Vallittu, Sufyan Garoushi, Lippo Lassila, Pekka K. Vallittu

**Affiliations:** a Department of Biomaterials Science and Turku Clinical Biomaterial Center –TCBC, Institute of Dentistry, University of Turku, Turku, Finland; b The Wellbeing Services County of Southwest Finland, Turku, Finland

**Keywords:** Composite–composite interface, delayed placement, interlayer adhesion, shear bond strength, oxygen inhibition layer

## Abstract

**Objective:**

To investigate the effect of delay time in resin composite placement and resin matrix composition on interlayer adhesion between two successive composite layers.

**Materials and methods:**

Three experimental resin composites were prepared by mixing 79 wt.% particulate filler with 21 wt.% dimethacrylate-based resin matrices (urethane dimethacrylate/triethylene glycol dimethacrylate [UDMA/TEGDMA]; ethoxylated bisphenol-A-dimethacrylate/urethane dimethacrylate/triethylene glycol dimethacrylate [Bis-EMA/UDMA/TEGDMA]; bisphenol A-glycidyl methacrylate/triethylene glycol dimethacrylate [Bis-GMA/TEGDMA]). Fifteen groups of specimens were prepared (*n* = 6/group), consisting of a cured composite substructure onto which a successive surface layer was applied at different time intervals following light-initiated polymerization (0, 10 minutes, 1, 24, and 72 hours). After 2 days of dry storage at 37°C, interlayer adhesion was evaluated by measuring shear bond strength (SBS) using a universal testing machine. Failure modes were visually assessed. In addition, oxygen inhibition layer (OIL) thickness was measured microscopically, and Vickers hardness and viscosity were determined. Data were analyzed using two-way and one-way analysis of variance followed by Tukey Tukey’s Honestly Significant Difference (HSD) tests (α = 0.05).

**Results:**

SBS values ranged from 10.3 ±2.4 to 24.5 ±3.0 MPa. Both placement delay time and resin matrix composition significantly affected SBS (*p* < 0.05). SBS decreased significantly when the application of the second successive layer was delayed beyond 1 hour, with the lowest values observed after 72 hours, particularly in Bis-GMA-based composites. The highest SBS was observed in the UDMA/TEGDMA group at 1 hour. Cohesive failure within the substructure was seen in all specimens. The UDMA/TEGDMA group exhibited the lowest OIL thickness (29.35 ± 0.69 µm).

**Conclusion:**

Both incremental application timing and resin matrix composition significantly influenced composite interlayer adhesion. Short delays (10–60 minutes) enhanced interlayer adhesion, whereas prolonged delays (24–72 hours) resulted in a reduction in interlayer adhesion.

## Introduction

Resin composites are widely used in restorative dentistry due to their versatility and continuous advancements in material properties. These materials consist of a polymer matrix reinforced with inorganic fillers, where the interaction at the filler–matrix interface, often mediated by silane coupling agents, plays a critical role in determining overall performance [1]. Modifications in filler characteristics and resin composition have enabled significant improvements in mechanical, physical, and esthetic properties, allowing resin composites to meet the diverse demands of clinical applications [2].

Most dental resins are primarily composed of dimethacrylate-based monomers, which have been extensively studied. Commonly used monomers include bisphenol A-glycidyl methacrylate (Bis-GMA), ethoxylated bisphenol-A-dimethacrylate (Bis-EMA), and urethane dimethacrylate (UDMA). Due to their relatively high viscosity, diluent monomers such as triethylene glycol dimethacrylate (TEGDMA) are often incorporated to improve handling characteristics [3]. Variations in monomer composition may influence polymerization behavior, network formation, and ultimately interlayer adhesion.

Despite several advances, conventional resin composites still present limitations, particularly related to polymerization shrinkage and limited depth of cure, which necessitate the use of an incremental layering technique [4, 5]. In such restorations, the integrity of the interface between successive composite layers is crucial, as inadequate interlayer adhesion may compromise the mechanical stability and longevity of the restoration [6]. During light polymerization, exposure to atmospheric oxygen leads to the formation of an oxygen inhibition layer (OIL), characterized by a resin-rich, partially polymerized surface [7–10]. This layer is thought to facilitate chemical interaction between increments through the formation of interpenetrating polymer networks. However, the influence of the OIL on interlayer adhesion remains controversial, with some studies reporting improved adhesion [7, 11, 12], while others demonstrate no significant effect [9, 13, 14].

In clinical practice, composite increments are typically applied immediately after curing the previous layer in direct restorations. However, delayed incremental layering may occur in several clinically relevant situations. In direct restorations, interruptions during the procedure may result in short delays between increments. In indirect or semi-direct techniques, restorations fabricated chairside may require additional composite application after try-in, proximal contact adjustment, or occlusal refinement prior to final cementation with resin composite luting materials. Furthermore, repair or modification of existing composite restorations at subsequent appointments represents a scenario involving prolonged delays between composite applications.

Previous studies have investigated the effect of delayed composite placement on interlayer adhesion, with inconsistent findings depending on factors such as surface condition, applying adhesive layer, aging, and material composition [15–17]. These discrepancies highlight the need for further investigation, particularly regarding the combined influence of delay time and resin matrix composition on interlayer adhesion. As the composite surface ages, the availability of free radicals decreases, potentially reducing the capacity for chemical interaction between layers. In addition, interlayer adhesion may be influenced by the chemistry of the resin matrix [18] and environmental factors such as moisture contamination [19]. However, the interaction between delay time and resin composition has not been fully elucidated.

Therefore, the aim of this study was to investigate the effect of delay time between composite layer placements and resin matrix composition on interlayer adhesion between successive layers of the same material. The null hypothesis was that neither delay time nor resin matrix would significantly affect interlayer adhesion.

## Materials and methods

### Materials

Bis-GMA and Bis-EMA were purchased from Esstech Inc. (Essington, PA, USA). UDMA, TEGDMA, camphoroquinone (CQ), and 2-(dimethylamino)ethyl methacrylate (DMAEMA) were obtained from Sigma-Aldrich Co. (St Louis, MO, USA). Silanated glass (barium silicate based) filler particles (Ø 0.7 µm) were received from Schott AG (UltraFine, Schott, Landshut, Germany). All of the reagents were used without purification.

### Preparation of resin composites

**Figure 1:**
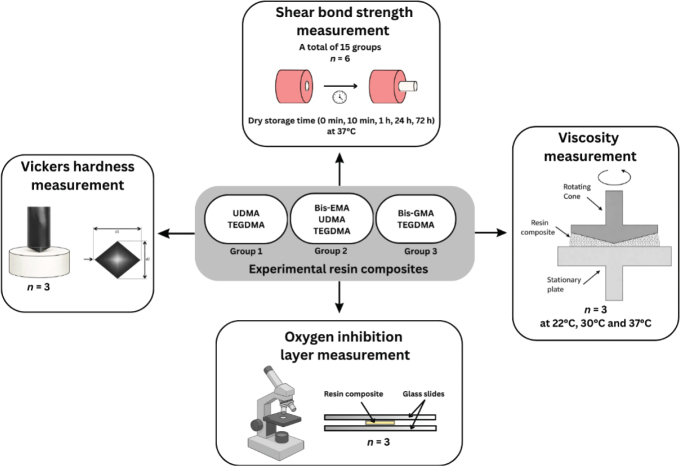
Schematic representation of the experimental workflow and the different test set-ups applied in the study. Bis-GMA: bisphenol A-glycidyl methacrylate; Bis-EMA: ethoxylated bisphenol-A-dimethacrylate; UDMA: urethane dimethacrylate; TEGDMA: triethylene glycol dimethacrylate.

This study focuses on three experimental resin composite materials differing in monomer composition (Table 1), each containing 0.7 wt.% CQ and 0.7 wt.% DMAEMA as the photoinitiator system. The experimental resin composites were prepared by mixing 79 wt.% of particulate filler with 21 wt.% of different combinations of dimethacrylate-based resin matrices. The mixing was carried out by using a high-speed mixing machine for 2 minutes (Hauschild Speed Mixer DAC 400.1, 3500 rpm). Subsequently, the experimental resin composites were evaluated under different test setups, as illustrated in Figure 1.

**Table 1:** Monomer composition of the studied groups.

Group	Monomers	wt.%
1	UDMA	70
	TEGDMA	30
2	Bis-EMA	35
	UDMA	35
	TEGDMA	30
3	Bis-GMA	70
	TEGDMA	30

Bis-GMA: bisphenol A-glycidyl methacrylate; Bis-EMA: ethoxylated bisphenol-A-dimethacrylate; UDMA: urethane dimethacrylate; TEGDMA: triethylene glycol dimethacrylate.

### Specimen preparation and shear bond strength testing

Cylindrical auto-polymerizing acrylic resin blocks (*n* = 90) were fabricated using a powder–liquid system (Self-Curing, Vertex Dental B.V., Netherlands), mixed at a ratio of 1.7 g powder (polymethyl methacrylate; PMMA) to 1 mL liquid (methyl methacrylate; MMA). A cylindrical cavity (7 mm in diameter and 4 mm in depth) was drilled into each block. These acrylic blocks served solely as molds or jigs (Figure 1) for the preparation and stabilization of specimens used in shear bond strength (SBS) testing.

**Figure 2:**
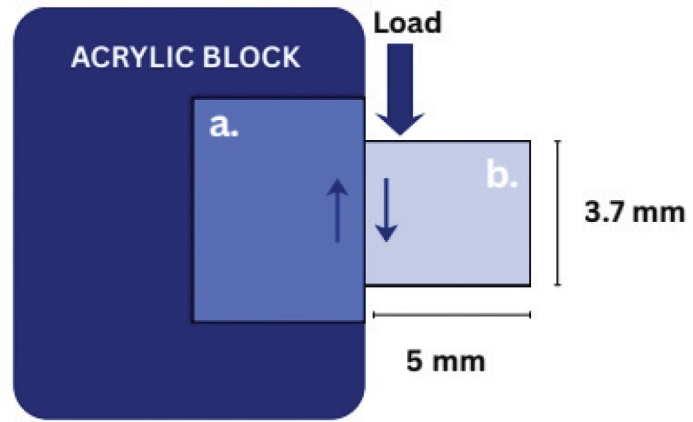
Schematic drawing of specimen preparation and the shear bond strength test setup. Shear stress (arrows) at the adhesive interface generated during loading. (a) Substructure cured composite and (b) second successive surface composite.

SBS was assessed between a substructure and a surface layer, both made from the same resin composite. The uncured resin composite designated as the substructure was placed into the acrylic block holes (Figure 2) and light-cured for 20 seconds per layer (2 mm) using an LED curing unit (Elipar S10, 3M ESPE, St. Paul, MN, USA). The curing device’s light tip was 1 mm away from the resin composite. The light intensity was 1200 mW/cm^2^, with a wavelength ranged from 400 to 480 nm (Marc Resin Calibrator, BlueLight Analytics Inc., Canada). After polymerization of the substructure, the successive surface layer was applied at different time intervals (0, 10 minutes, 1, 24, and 72 hours). During the waiting period, the substructure specimens were stored dry in an incubator at 37°C.

The successive surface layer was applied using a plastic mold with an inner diameter of 3.7 mm and a height of 5.0 mm (Figure 2), positioned directly on top of the cured substructure without the use of any adhesive. The surface layer was light cured for 20 seconds from the top and for a total of 40 seconds from the sides (20 seconds from each side). In total, 15 groups of specimens were prepared (*n* = 6/group).

After 2 days of dry storage at 37°C, SBS was measured using a universal testing machine (Model LRX; Lloyd Instruments Ltd, AMETEK, West Sussex, UK), with a shear load applied at the adhesive interface at a crosshead speed of 1 mm/min. The shear force was applied parallel to the adhesive interface (Figure 2) to ensure a comparable stress distribution across all specimens. The maximum force (N) at failure was recorded, and bond strength (MPa) was calculated by dividing this value by the bonding area (mm^2^) using the formula:


$$SBS\left(MPa\right)=\frac{Maximum\;Load\;\left(N\right)}{Bonding\;area\;\left(mm^{2}\right)}$$

Failure mode analysis was performed by two examiners through visual inspection under standardized lighting conditions following testing.

### Viscosity measurement

The viscosity of the resin composite materials was determined using a parallel plate rheometer (RheoStress 300, Thermo Haake, Thermo Electron, Karlsruhe, Germany). Uncured resin composite was placed on the lower plate. The upper plate, with a diameter of 20 mm, was then lowered on top of the lower plate so that the gap between the plates was set to 0.5 mm. Before taking the measurements, any excess resin composite was removed from the sides of the plates. All measurements were performed at three different temperatures (22, 30, and 37°C) for 300 seconds and were repeated three times for each temperature (*n* = 3/group). Viscosity was calculated from the rheological measurement with an equation η = σ/γ (η = viscosity, σ = shear stress, and γ = shear stress).

### Vickers hardness measurement

To determine the Vickers hardness of each resin composite, three groups of specimens were prepared (*n* = 3/group). The uncured resin composite was placed into a cylindrical plastic mold with an inner diameter of 8.6 mm and a height of 7.5 mm. It was light-cured for 20 seconds for each 2-mm increment from the top, followed by 40 seconds of additional curing from the sides (20 seconds per side). After polymerization, the specimen surfaces were polished using silicon carbide grinding paper (grit 4000; SiC, Struers, Copenhagen, Denmark) on an automatic grinding machine with water cooling (LaboPol-25, Struers A/S, Copenhagen, Denmark).

The Vickers hardness of the polished surfaces was measured using a microhardness tester (Rtec, Rtec Instruments Inc., USA) equipped with a Vickers diamond indenter in accordance with ISO 6507. Ten indentations were made on each specimen using a 3 N load and a 30 seconds dwell time, with each indentation spaced 0.5 mm apart.

### Oxygen inhibition layer measurement

The OIL of each resin composite was optically measured. Three specimens were prepared for each resin composite (*n* = 3/group). The material was applied onto a glass microscope slide positioned between two glass spacers with a thickness of 0.10 mm. A second glass slide was placed on top of the spacers, and the composite was light-cured for 40 seconds. The depth of the oxygen-inhibition layer was measured at five different points on each specimen using a Leica DMLB microscope (Leica, Wetzlar, Germany) at ×20 magnification with a calibrated micrometer disk. Images of the inhibition layer were captured using a computer imaging program (Toupview, Hangzhou ToupTek Photonics Co., Zhejiang, China).

### Statistical analysis

Statistical analysis was performed using SPSS software (Version 29.0, IBM Corp., Armonk, NY, USA). Two-way analysis of variance (ANOVA) was used to evaluate the effects of delay time and resin matrix composition on interlayer adhesion (SBS) and the effects of material and temperature on viscosity. One-way ANOVA was applied for comparisons within individual groups where appropriate post hoc pairwise comparisons were performed using Tukey’s test. The level of statistical significance was set at α = 0.05.

## Results

**Figure 3:**
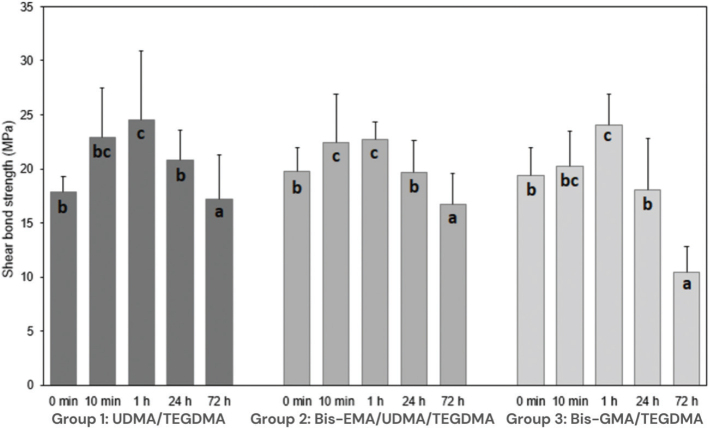
Shear bond strength (SBS) means and standard deviations for the three resin composite groups at different delay times between layer applications. Different letters indicate statistically significant differences (*p* < 0.05) among the groups. Bis-GMA: bisphenol A-glycidyl methacrylate; Bis-EMA: ethoxylated bisphenol-A-dimethacrylate; UDMA: urethane dimethacrylate; TEGDMA: triethylene glycol dimethacrylate.

**Figure 4:**
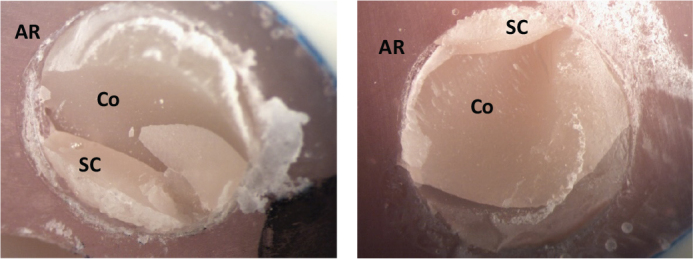
Representative photographs showing cohesive failure within the composite substructure layer after shear bond strength testing. Structures pictured: AR (acrylic resin), SC (substructure composite), and Co (cohesive failure).

The mean SBS values for all specimens ranged from 10.3 ± 2.4 MPa to 24.5 ± 3.0 MPa (Figure 3). Two-way ANOVA revealed that both delay time and resin matrix composition had a significant effect on SBS values (*p* < 0.05). In all three resin composite groups, SBS was highest when the second layer was applied within 10 minutes to 1 hour (Figure 3); however, these differences were not statistically significant in all groups (*p* > 0.05). Delayed application for 72 hour resulted in significantly lower SBS values in all groups (*p* < 0.05). Group 1 (UDMA/TEGDMA) and Group 2 (Bis-EMA/UDMA/TEGDMA) exhibited relatively similar trends, whereas Group 3 (Bis-GMA/TEGDMA) showed a more pronounced decrease at 72 hour. Cohesive failure within the substructure layer was observed in all specimens (Figure 4).

**Figure 5:**
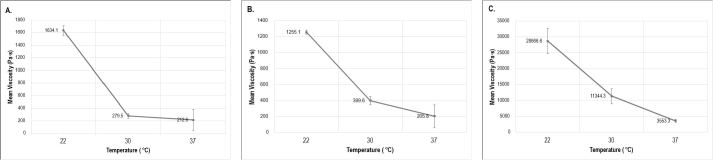
Means and standard deviations of viscosities (Pascal-second) at three different temperatures of studied resin composites. A (UDMA/TEGDMA), B (Bis-EMA/UDMA/TEGDMA) and C (Bis-GMA/TEGDMA). Bis-GMA: bisphenol A-glycidyl methacrylate; Bis-EMA: ethoxylated bisphenol-A-dimethacrylate; UDMA: urethane dimethacrylate; TEGDMA: triethylene glycol dimethacrylate.

Mean viscosities of each resin composite at the three tested temperatures are presented in Figure 5. As expected, viscosity decreased with increasing temperature for all materials (*p* < 0.05). The Bis-GMA/TEGDMA group exhibited the most pronounced decrease in viscosity between 22 and 37°C. In contrast, Bis-EMA/UDMA/TEGDMA and UDMA/TEGDMA showed similar viscosities across the three temperature points and were less sensitive to temperature elevation. Statistically, both material composition and temperature had a significant effect (*p* < 0.05) on viscosity values.

Vickers hardness testing revealed that Group 2 (Bis-EMA/UDMA/TEGDMA) had the highest hardness value (157.6 ± 4.8), followed by Group 3 (Bis-GMA/TEGDMA) at 135.6 ± 7.9, and Group 1 (UDMA/TEGDMA) showing the lowest value (117.8 ± 3.9), respectively (*p* < 0.05).

**Figure 6:**
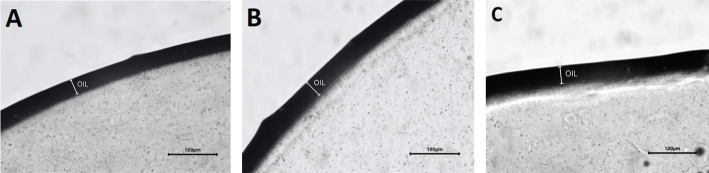
Microscopy images of the oxygen inhibition layer (OIL) in UDMA/TEGDMA (A), Bis-EMA/UDMA/TEGDMA (B) and Bis-GMA/TEGDMA (C). Scale bar = 100 µm. Bis-GMA: bisphenol A-glycidyl methacrylate; Bis-EMA: ethoxylated bisphenol-A-dimethacrylate; UDMA: urethane dimethacrylate; TEGDMA: triethylene glycol dimethacrylate.

The optically measurable thickness of the OIL was determined for the three resin composites (Figure 6). Group 2 (Bis-EMA/UDMA/TEGDMA) exhibited the highest mean OIL thickness (36.03 ± 0.53 µm), followed by Group 3 (Bis-GMA/TEGDMA) with 34.16 ± 0.49 µm, and Group 1 (UDMA/TEGDMA) showing the lowest thickness (29.35 ± 0.69 µm). One-way ANOVA indicated a statistically significant difference in OIL thickness among the groups (*p* < 0.05).

## Discussion

The incremental layering technique is recommended to reduce polymerization shrinkage stress and improve the depth of cure of conventional resin composites [20]. Consequently, interlayer adhesion between successive layers becomes a critical factor influencing the clinical performance of these restorations. The present study aimed to investigate the effect of delay time between composite layer placements and resin matrix composition on interlayer adhesion, assessed by measuring SBS between successive layers of the same material. The null hypothesis stated that there would be no significant differences in interlayer adhesion among the tested groups. However, the results demonstrated that both the timing of incremental application and the resin matrix composition significantly affected interlayer adhesion, leading to rejection of the null hypothesis.

The notably high SBS values observed when the second layer was applied within 10 minutes to 1 hour may be explained by several interacting mechanisms. During this early post-cure phase, residual free radicals and unreacted methacrylate groups remain within the OIL and near-surface zone of the first increment [12]. These reactive species can copolymerize with fresh monomers in the second layer, creating an interdiffusion zone with substantial chemical cross-linking [21]. This reaction-driven integration effectively ‘welds’ the layers together and likely accounts for the increased bond strengths and cohesive failure type. In addition, the first increment may not have reached its maximum degree of post-cure conversion, so the surface remains sufficiently viscoelastic (i.e. not yet fully vitrified) to better accommodate the new layer. This may reduce interfacial stress concentration and microvoid formation, further improving interlayer adhesion [22]. In contrast, when application of the second layer was delayed by 24–72 hours (corresponding to restoration modification or repair at subsequent appointments), several mechanisms may account for the significant reduction in SBS observed in this study. Over time, reactive sites may diminish as free radicals terminate and unreacted methacrylate groups might undergo slow post-cure conversion or other secondary reactions, thereby reducing the potential for chemical bonding with the fresh layer [23]. The surface also may become more rigid and less wettable, which could limit monomer diffusion and interpenetration. Additionally, any oxygen-inhibited superficial layer may age, oxidize, or lose its inherent tackiness, potentially compromising intimate contact and hindering the formation of a strong interfacial network [24].

Interestingly, despite the reduction in SBS after delayed application, all specimens in this study exhibited cohesive failure within the underlying composite. This failure pattern might suggest that some degree of monomer interpenetration or interfacial integration still occurred even after a 72-hour delay. This finding may align with the observations of Dall’Oca et al., who reported that a substantial decline in bond strength between an existing and a fresh composite may require more than 14 days to become evident [14]. Contrasting reports in the literature indicate that composite repair bonds may actually be stronger when the substrate composite lacks an OIL [13, 25, 26]. These authors suggest that the OIL may contain reduced concentrations of photoinitiator and may undergo incomplete polymerization, potentially creating a structurally weaker interfacial zone.

Despite the conventional expectation that immediate layering with a fresh OIL would yield the highest bond strength and in line with findings from some earlier studies [26, 27], the SBS measured at 0 minute in this study was lower than that obtained after short delays (Figure 3). Several material-related and practical factors may account for this behavior. Immediately after light curing, the surface layer may still be undergoing rapid polymerization kinetics and structural relaxation, creating transient instability that affects the adaptation of the subsequent increment. The freshly cured surface may also not yet achieve optimal wettability and may present temporary micro-irregularities or residual polymerization-shrinkage stresses that hinder intimate contact with the new layer. Another possible explanation is that the concentration of photoinitiator or free radicals at the surface becomes rapidly depleted during the immediate post-cure phase, resulting in fewer reactive species available for efficient interlayer copolymerization. These possible combined factors may contribute to the slightly lower interlayer adhesion at the immediate time point despite the presence of an OIL.

The consistently higher SBS values observed in Group 1 (UDMA/TEGDMA) compared with the other two composites can be attributed to the chemical structure and network dynamics of UDMA-based matrices. UDMA is known to have lower viscosity, higher flexibility, and more mobile polymer chains than Bis-GMA-rich systems [28–30]. These features promote more effective interpenetration and copolymerization between increments, resulting in a more cohesive interlayer network. Furthermore, UDMA tends to undergo a slower vitrification process during light curing, meaning that a greater fraction of unreacted double bonds and mobile radicals remain available for reaction when the subsequent layer is applied [29]. This chemical ‘reactivity reserve’ likely explains why UDMA/TEGDMA maintained strong interlayer adhesion even when delay times were extended.

Group 3 (Bis-GMA/TEGDMA) showed the most pronounced drop in SBS, particularly at 72 hours, indicating that Bis-GMA-dominant matrices are more susceptible to the effects of delayed layering. Bis-GMA’s high viscosity and rigid aromatic backbone restrict monomer mobility and interlayer diffusion, and once polymerized, its surface rapidly vitrifies, reducing the availability of reactive sites [28, 31]. With increasing delay, additional post-curing and surface oxidation may further limit the potential for copolymerization with a fresh increment. Notably, these same properties may explain why the Bis-GMA group showed the highest SBS at 0 minute: immediately after curing, Bis-GMA forms a relatively thick, monomer-rich OIL due to slower surface polymerization, providing an exceptionally reactive interface for instant layering [32, 33]. Once this short reactive phase passes, however, Bis-GMA’s stiff network and rapid surface vitrification sharply reduce its bonding potential, resulting in the observed decline at longer delays.

Consistently, the thickness of the OIL reflected the influence of resin chemistry: Group 2 (Bis-EMA/UDMA/TEGDMA) exhibited the thickest OIL, followed by Group 3 (Bis-GMA/TEGDMA), while Group 1 (UDMA/TEGDMA) produced the thinnest layer. According to Pereira et al. [32], Bis-EMA-based monomers behave similarly to Bis-GMA-based monomers in terms of degree of monomer conversion and curing potential as a function of depth, which helps explain the comparable OIL thickness observed for Groups 2 and 3. As previously discussed, UDMA monomers have more mobile polymer chains, facilitating higher surface monomer conversion and resulting in a thinner OIL. It is important to note that differences in material formulations and specimen preparation among studies limit the possibility of directly comparing the present OIL values with previously published data.

From a bonding perspective, in our study, the thickness of the OIL appeared to correlate with the SBS measured after immediate application (0 minute), as Group 1 (UDMA/TEGDMA) showed the lowest bond strength at this time point (Figure 3). These findings suggest that OIL behavior is not universally beneficial or detrimental; rather, its effect is material dependent and closely related to the properties of the underlying monomer system.

To ensure consistency across all experimental groups, standardized light-curing conditions were applied throughout the study. All specimens were polymerized using the same hand light-curing unit under identical irradiation time, intensity, and positioning. This standardization was essential to minimize variability in polymerization kinetics and to allow direct comparison of interlayer adhesion between groups. It is well established that curing parameters influence the degree of conversion and the characteristics of the OIL, which in turn may affect interlayer adhesion [34]. Unlike several previous studies, this study did not use an intermediate adhesive resin during test specimen fabrication [35, 36]. Typically, intermediate adhesive resins have the same monomer composition as the resin composites they are designed to bond but additional solvent and even surface dissolution containing intermediate resins were also studied [37, 38]. The absence of fillers in intermediate adhesive resins results in lower viscosity compared to their filler-containing composite counterparts. This lower viscosity enhances surface wettability and may improve the diffusion of monomers to the substrate. Although the use of intermediate adhesive resin is common in the repair of old composite restorations or when indirectly made restorations are luted, it is not recommended for use in the direct incremental filling technique.

Vickers hardness testing after polishing revealed a different trend, with Group 2 (Bis-EMA/UDMA/TEGDMA) exhibiting the highest hardness values, followed by Group 3 (Bis-GMA/TEGDMA), while Group 1 (UDMA/TEGDMA) showed the lowest. These findings reflect intrinsic differences in resin matrix stiffness and network rigidity. They partially support our previous explanation, as UDMA/TEGDMA demonstrated superior SBS after short delays despite its lower hardness, consistent with a more flexible and less densely cross-linked polymer network. This enhances copolymerization during layering, contributing to the observed interlayer bond strength.

Viscosity measurements at different temperatures further elucidated the behavior of these materials under clinically relevant conditions. As expected, all materials exhibited decreasing viscosity with increasing temperature [39]. The Bis-GMA/TEGDMA resin composite showed the most pronounced viscosity reduction between 22 and 37°C, consistent with its high initial molecular weight and inherently rigid backbone; once heated, molecular mobility increases substantially, allowing a marked drop in viscosity [40]. In contrast, the Bis-EMA/UDMA/TEGDMA and UDMA/TEGDMA groups demonstrated less temperature sensitivity, reflecting more flexible monomer systems with lower baseline viscosity. These differences in flow behavior complement the previous observations on SBS: during specimen preparation at room temperature, the high viscosity of Bis-GMA-based resin (Group 3) may have limited intimate adaptation or wettability at the interface, partially contributing to the lower SBS values measured in this study.

Overall, the results indicate that resin composition, handling conditions, and application timing critically influence composite interlayer adhesion. Clinically, these findings underscore the importance of timely incremental placement and careful selection of resin matrix composition to optimize the performance and long-term durability of composite restorations.

This study has several limitations. First, only three methacrylate-based resin composite formulations were evaluated, which may not represent the full range of contemporary restorative materials, such as ormocer-based systems. Second, no adhesive intermediate layer was used, which may limit direct clinical applicability, particularly in repair procedures where adhesive systems are commonly employed. Although the SBS test is widely used, it is sensitive to specimen geometry, loading direction, and stress concentration, which may lead to over- or underestimation of the true interfacial strength. A microtensile bond strength (µTBS) test could provide a more uniform stress distribution and potentially a more reliable assessment of the interfacial region; however, this method is technically demanding and prone to premature failures, particularly in brittle composites.

Furthermore, although the experimental conditions were designed to simulate certain clinical scenarios, this in vitro study does not fully replicate the complexity of the oral environment. In particular, delayed application conditions did not account for factors such as humidity, saliva contamination, or intraoral aging, which may influence interlayer adhesion. Nevertheless, the controlled laboratory setup allowed systematic evaluation of the variables under investigation. Finally, no formal sample size calculation was performed; the sample size was based on previous similar studies reported in the literature, which may represent a methodological limitation.

Future studies should further explore factors that may influence interlayer adhesion beyond those investigated in the present work. In particular, the role of photoinitiator systems, including variations in type and concentration, should be examined. In this context, direct assessment of free radical generation and decay may provide additional insight into the mechanisms governing interlayer adhesion

## Conclusion

Within the limitations of this study, the results demonstrate that both incremental application timing and resin matrix composition significantly influence composite interlayer adhesion. From a clinical perspective, applying successive composite layers within a short time interval (10–60 minutes) may enhance interlayer adhesion, whereas prolonged delays (24–72 hours) should be avoided, particularly with Bis-GMA-based composites. It should be noted that these findings are specific to the tested resin formulations and may not be directly generalizable to all commercially available composite materials.

## Data Availability

The datasets used and/or analyzed during the current study available from the corresponding author on reasonable request.
